# Exploring the potential pharmacodynamic material basis and pharmacologic mechanism of the *Fufang-Xialian-Capsule* in chronic atrophic gastritis by network pharmacology approach based on the components absorbed into the blood

**DOI:** 10.1098/rsos.171806

**Published:** 2018-06-13

**Authors:** Shizhe Li, Tengfei Xu, Shu Liu, Zhiqiang Liu, Zifeng Pi, Fenrui Song, Yongri Jin

**Affiliations:** 1 National Center of Mass Spectrometry in Changchun and Jilin Province Key Laboratory of Chinese Medicine Chemistry and Mass Spectrometry and Chemical Biology Laboratory, Changchun 130022, People's Republic of China; 2 State Key Laboratory of Electroanalytical Chemistry, Changchun Institute of Applied Chemistry, Chinese Academy of Sciences, Changchun 130022, People's Republic of China; 3 College of Chemistry, Jilin University, Changchun 130012, People's Republic of China

**Keywords:** *Fufang-Xialian-Capsule*, chronic atrophic gastritis, UPLC-Q-TOF-MS, absorbed components into blood, network pharmacology

## Abstract

In this study, a new network pharmacology approach based on the components absorbed into the blood was used to investigate the pharmacodynamic material basis and the pharmacologic mechanism of the *Fufang-Xialian-Capsule* (*FXL*) in treating chronic atrophic gastritis (CAG). Initially, we confirmed the components absorbed into the blood by ultra-high-performance liquid chromatography coupled with quadrupole time-of-flight mass spectrometry. Then, the network approach, which was based on the results of components absorbed into the blood, was used to analyse the pharmacodynamic material basis and the pharmacologic mechanism of *FXL* on treating CAG. As a result, 22 absorbed components were found in rat plasma. Given the results of the absorption analysis of the components, eight pathways associated with CAG development were found. The targets linked to these pathways are the drug targets of *FXL* in CAG treatment. The components associated with these targets are the potential pharmacodynamic material basis and exert synergy in regulating pathways during CAG treatment.

## 1. Introduction

Chronic atrophic gastritis (CAG) is a common digestive system disease, which has a high risk of causing gastric cancer [1]. CAG is usually characterized by an atrophic gastric mucosa, reduced gastric acid secretion and pathologically altered epithelium along with the intestinal epithelium [2]. The main cause of CAG includes *Helicobacter pylori* infection [3], autoimmune factors [4], vitamin B12 deficiency [5] and gastric mucosal lesions [2]. Given the high risk of gastric cancer in CAG, suitable treatment for this condition is urgently needed.

The *Fufang-Xialian-Capsule* (*FXL*) is a traditional Chinese medicine (TCM) formula for treating CAG that is improved from *Banxia Xiexin Decoction* (*BXD*). *BXD* was first recorded in the Treatise on Febrile Diseases (Shang Han Lun) written by Zhang Zhongjing and is still used to date [6]. *BXD* is a classical formula that has long been used to treat chronic gastrointestinal diseases, such as chronic gastritis, chronic intestinal gastritis and peptic ulcer disease [7]. However, *BXD* is also widely used in clinical treatment of chronic gastrointestinal disease nowadays [8–10]. Some reports exist as regards the pharmacologic research [11] and pharmacokinetic research [12] on *BXD.* However, given the modified formula for BXD, no reports have emerged about *FXL*.

The formula for *FXL* includes *Rhizoma Pinelliae Preparata*, *Coptis chinensis*, *Rhizoma Zingiberis*, *Panax ginseng*, *Scutellaria baicalensis*, *Magnolia officinalis*, *Olibanum* and *Glycyrrhiza Radix Preparata*. The chemical constitutions of *FXL* were clarified by our previous work; the main components include flavones, flavone glycosides, ginsenoside, lignins, triterpene saponins, flavanones, chalcones, coumarin and alkaloids [13]. However, the absorbed bioactive component and the pharmacologic mechanism of *FXL* remain unclear. Many reports have emerged about the network pharmacology approach, which uses some databases to investigate the pharmacodynamic compounds and pharmacological mechanisms of TCM [14,15]. Even so, the traditional network pharmacology approach usually uses databases to collect TCM constituents as candidate pharmacodynamic compounds [16,17]. This strategy yields a broad candidate range that leads to a difficulty in directly clarifying pharmacodynamic components and pharmacologic mechanisms. In recent years, an increasing number of reports have arisen regarding the absorbed bioactive components of TCM *in vivo* [18,19]. These components are absorbed through the stomach and then distributed in target organs by blood transport [20]. Thus, the real pharmacodynamic components can be obtained from the components absorbed into the blood.

In this study, a new network pharmacology approach based on the absorbed components into the blood was used to screen for potential pharmacodynamic components. Initially, ultra-high-performance liquid chromatography coupled with quadrupole time-of-flight mass spectrometry (UPLC-Q-TOF-MS) was used to detect the absorbed components of *FXL* in rat plasma. The absorbed components were identified on the basis of our previous work on material basis. After the identification, the compound targets of the absorbed components were collected by database, and the compounds related to CAG were selected. Then, the pathways associated with these targets were also selected, and the networks of compound target pathway were built. Through the components absorbed into the blood and network pharmacology approach, we could realize the pharmacodynamic material approach and explain directly the relationship of drug, target and disease. The results could also provide basis for further research on the potential pharmacological and molecular mechanisms of *FXL* in treating CAG. This method could be used to investigate other Chinese medicine formulae.

## 2. Experimental set-up

### 2.1. Materials and reagents

High-performance liquid chromatography-grade methanol, acetonitrile and formic acid were purchased from Fisher Scientific (Loughborough, UK). Ultrapure water was prepared by a Milli-Q plus (Millipore, MA, USA) water purification system. *FXL* was manufactured in our laboratory, and the batch used in our previous research was applied.

### 2.2. Components absorbed into the blood

#### 2.2.1. Animals

Male Sprague-Dawley rats (220 ± 20 g) were purchased from the Experimental Animal Center of Jilin University. The rats were housed (five per cage) under a 12 L : 12 D cycle for 5 days to adapt to the environment. All rats were given free access to chow and tap water. The temperature and relative humidity of the animal room were maintained at 22 ± 2°C and 60%, respectively. The experiments and procedures were compliant with the principles of laboratory animal use and care. The study was approved by the Institutional Animal Ethics Committee of Jilin University.

#### 2.2.2. Preparation of plasma

A total of 30 rats orally administered with *FXL* (3 g kg^−1^ body weight) were used to collect medicated plasma samples, and five rats orally administered with deionized water were used to obtain blank plasma samples. All the rats were dosed daily for 5 days.

The blood samples were collected at 0.5, 1, 2, 4, 6 and 8 h, respectively, after the last dose (*n* = 5 for each group) with tubes processed by 0.1% HPMC-Na solution. The blood was centrifuged at 3000 r.p.m. for 5 min to obtain plasma under 4°C, and the plasma of each groups was combined and stored at −80°C. Next, 1 ml of the obtained plasma was mixed with 5 ml of organic solution (methanol and acetonitrile 3 : 2, v/v), whirled for 5 min and then centrifuged at 8000 r.p.m. for 10 min. The supernatant was transferred to a clean tube and dried under a gentle flow of nitrogen gas at 30°C. The residue was dissolved with 200 µl of methanol and centrifuged at 10 000 r.p.m. for 10 min. The supernatant was collected, and 5 µl was injected for UPLC-Q-TOF-MS analysis.

#### 2.2.3. UPLC-Q-TOF-MS analysis

The analysis was performed using a Waters Acquity UPLC system coupled with a Q-TOF SYNAPTG2 high-definition mass spectrometer (Waters, USA). A waters acquity UPLC BEH C18 column (1.7 µm, 2.1 mm × 50 mm) was used to separate the samples. The column temperature was 30°C, and the sample injection volume was 5 µl. Acetonitrile (A) and 0.1% (v/v) formic acid (B) were used as mobile phases. Gradient elution with a flow rate of 300 µl min^−1^ was performed as follows: 5–15% A at 0–5 min, 15–30% A at 5–12 min, 30–50% A at 12–18 min, 50–80% A at 18–40 min, 80–100% A at 40–50 min, 100% A at 50–52 min in negative ion mode and 5–20% A at 0–3 min, 20–40% A at 3–7 min, 40–60% A at 7–13 min, 60–100% at 20–22 min and 100% A at 22–23 min in positive ion mode.

The analysis was performed using an electrospray ionization source in positive and negative ion modes; full scan mode was selected, and the mass range was *m/z* 100–1300 Da. Nitrogen was applied as cone and desolvation gas, with flow rates of 50 and 700 l h^−1^, respectively. In positive ion mode, the capillary, cone and extraction voltages were 3.0, 40 and 4.0 kV, respectively. In negative ion mode, capillary, cone and extraction voltages were 2.5, 40 and 4.0 kV, respectively. Leucine enkephalin (*m/z* 556.2771 in positive ion mode and *m/z* 554.2615 in negative ion mode) was used as reference mass. Sodium formate was used to set up a mass spectrometer calibration in positive and negative ion modes.

### 2.3. Network pharmacology approach

#### 2.3.1. Compound target of *Fufang-Xialian-Capsule*

On the basis of the results of absorbed components into blood, the targets associated with these compounds were collected through databases, such as HIT (lifecenter.sgst.cn/hit/) and STITCH (stitch1.embl.de) [20]. By inputting all molecular formulae of absorbed components into HIT and STITCH, we obtained the symbol of compound targets. After synthesizing the data from these databases, we obtained sufficient targets associated with absorbed components (electronic supplementary material, table S1).

#### 2.3.2. Chronic atrophic gastritis target

The genes associated with CAG were collected from DisGeNET (disgenet.org), which is a discovery platform. DisGeNET integrates on gene–disease associations from several public data sources and literature, so it provides more comprehensive information on gene–disease associations than other databases. We used CAG as keyword and created the selection. Through the search, we obtained a total of 113 genes (electronic supplementary material, table S2).

#### 2.3.3. Pathway analysis to explore the mechanisms of *Fufang-Xialian-Capsule* in chronic atrophic gastritis treatment

To explore the mechanisms of *FXL* in CAG, we investigated the pathways associated with CAG treatment. The genes associated with CAG were inputted into the DAVID database (david.ncifcrf.gov/home.jsp), and then *Homo sapiens* was selected as the species. We then attained the pathways associated with CAG. The KGEE PATHWAY database (genome.jp/kegg/pathway.html) was also used to collect the pathways associated with CAG treatment. By synthesizing the data from these databases, we obtained sufficient pathways associated with CAG treatment.

#### 2.3.4. Network construction

On the basis of the results of the absorbed components into the blood, a series of networks was built through the Cytoscape software. The networks were created as follows: (i) compound–compound target network of *FXL*, (ii) *FXL*-CAG target network and (iii) *FXL*-CAG network pathways.

## 3. Results and discussion

### 3.1. Absorbed components into the blood

The medicated plasma samples and blank plasma were analysed by UPLC-Q-TOF-MS. We then compared the total ion chromatograms of the medicated plasma with the blank plasma. Along with the identification results of a previous study [13], we found 22 absorbed components, including seven alkaloids, nine flavonoids, five ginsenosides and one triterpene (table 1), from *FXL*.

**Table 1.** Absorbed components in rat plasma. C, Coptidis Rhizoma; S, Scutellariae Radix; P, Ginseng Radix Et Rhizoma; G, Glycyrrhizae Radix Et Rhizoma Praeparata Cum Melle.

no.	Tr	observed *m/z* (+/−)	error	formula	MS/MS	identification	source
P1	3.01	342.1705	0	C_20_H_24_NO_4_	297,265	magnoflorine	C
P2	4.11	322.1076	0.3	C_19_H_16_NO_4_	307,294,279	berberrubine	C
P3	4.72	338.1383	−2.7	C_20_H_20_NO_4_	323,322,320,307,294	jatrorrhizine	C
P4	4.77	336.1228	−2.1	C_20_H_18_NO_4_	321,306,292	epiberberine	C
P5	4.84	320.0916	−1.9	C_19_H_14_NO_4_	292,277,264	coptisine	C
P6	5.46	352.1549	0.6	C_21_H_22_NO_4_	337,321,308	palmatine	C
P7	5.54	336.1239	1.2	C_20_H_18_NO_4_	321,306,292	berberine	C
P8	6.4	547.1447	−0.7	C_26_H_28_O_13_	487,457,427, 367,337	chrysin-6-c-ara-8-c-glu	S
P9	6.99	547.1451	0	C_26_H_28_O_13_	487,457,427, 367,337	chrysin-6-c-glu-8-c-ara	S
P10	9.68	845.4938	4.73	C_42_H_72_O_14_	799,637,475	ginsenoside Rf	P
P11	10.36	445.0766	−0.9	C_21_H_18_O_11_	269	apigenin-7-glucuronide	S
P12	10.60	459.0927	0	C_22_H_20_O_11_	283,268	oroxylinA-7-glucuronide	S
P13	10.83	475.0883	1.5	C_22_H_20_O_12_	299,284	5,6,7-trihydroxy-8-methoxy flavone-7-glucuronide	S
P14	11.09	445.0773	0.7	C_21_H_18_O_11_	269	baicalin	S
P15	11.24	459.0924	−0.6	C_22_H_20_O_11_	283,268	wogonoside	S
P16	13.68	1107.5999	4.3	C_54_H_92_O_23_	945	ginsenoside Rb1	P
P17	13.97	1123.5824	−6.8	C_53_H_90_O_22_	1077	ginsenoside Rc	P
P18	14.28	1123.5859	−3.6	C_53_H_90_O_22_	1077	ginsenoside Rb2	P
P19	14.89	991.5646	0.3	C_48_H_82_O_18_	945,783	ginsenoside Rd	P
P20	14.94	821.3959	0	C_42_H_62_O_16_	351,193	glycyrrhizin	G
P21	15.01	283.0606	1.0	C_16_H_12_O_5_	268	wogonin	S
P22	15.55	283.0611	0	C_16_H_12_O_5_	268	oroxylin A	S

#### 3.1.1. Alkaloids

Compound **1** showed [M]^+^ ion at *m/z* 342.1705 and fragment ions at *m/z* 297 and 265. The MS/MS fragmentation behaviours were similar to that of magnoflorine. Compound **2** revealed [M]^+^ ion at *m/z* 322.1076 and MS/MS fragment ions at *m/z* 307, 294 and 279; the fragmentation behaviours corresponded to berberrubine. Compound **3** presented a [M]^+^ ion at *m/z* 338.1383, and the fragment ions at *m/z* 323, 322, 320, 307 and 294 corresponded to [M-CH_3_]^+^, [M-CH_3_-H]^+^, [M-CH_3_-H-2H]^+^, [M-CH_3_-CH_3_-H]^+^ and [M-CH_3_-CO-H]^+^, respectively; the MS/MS fragmentation behaviours were similar to those of jatrorhizine. Compounds **4** and **7** individually showed [M]^+^ ions at *m/z* 336.1228 and 336.1239, respectively, and the MS/MS fragment ions at *m/z* 321, 306 and 292 corresponded to [M-CH_3_]^+^, [M-CH_3_-CH_3_]^+^ and [M-CH_3_-CH_3_-CH_2_]^+^, respectively. The MS/MS fragmentation behaviours were similar to epiberberine and berberine. Given our previous study, compounds **4** and **7** could be identified as epiberberine and berberine, respectively. Compound **5** showed [M]^+^ ions at *m/z* 320.0916, and the fragment ions at *m/z* 292, 277 and 264 corresponded to [M-CO]^+^, [M-CO-CH_3_]^+^ and [M-CO-CO]^+^, respectively. The MS/MS fragmentation behaviours were similar to those of coptisine. Compound **6** revealed a [M]^+^ ion at *m/z* 352.1549 and fragment ions at *m/z* 337, 321, and 308, which corresponded to [M-CH_3_]^+^, [M-CH_3_-CH_4_]^+^ and [M-CH_3_-CO-H]^+^, respectively. The MS/MS fragmentation behaviours were similar to those of palmatine.

#### 3.1.2. Flavonoids

Compounds **8** and **9** individually showed [M-H]^−^ ions at *m/z* 547.1447 and 547.1451, and all the fragment ions showed at *m/z* 487, 457, 427, 367 and 337; the MS/MS fragmentation behaviours were similar to chrysin-6-c-ara-8-c-glu and chrysin-6-c-glu-8-c-ara. Compared with the retention times of a previous study, compounds **8** and **9** could be identified as chrysin-6-c-ara-8-c-glu and chrysin-6-c-glu-8-c-ara. Compounds **11** and **14** showed [M-H]^−^ ions at *m/z* 445.0770 and 445.0773, and these two compounds showed the same fragment ions at *m/z* 269, which corresponded to [M-H-GluA]^−^; the MS/MS fragmentation behaviours were similar to baicalin and apigenin-7-glucuronide. By comparing the retention times with our previous study, compounds **11** and **14** could be identified as apigenin-7-glucuronide and baicalin, respectively. Compound **13** showed [M-H]^−^ ion at *m/z* 475.0883, and the fragment ions at *m/z* 299 and 284 corresponded to [M-H-Glu]^−^ and [M-H-Glu-CH_3_]^−^; the MS/MS fragmentation behaviours were similar to 5,6,7-trihydroxy-8-methoxyflavone-7-glucuronide. According to our previous study, we identified compound **13** as 5,6,7-trihydroxy-8-methoxyflavone-7-glucuronide. Compounds **12** and **15** showed [M-H]^−^ ions at *m/z* 459.0927 and 459.0924, and all the fragment ions individually showed *m/z* 283 and 268, which corresponded to [M-H-GluA]^−^ and [M-H-GluA-CH_3_]^−^, respectively. The MS/MS fragmentation behaviours were similar to oroxylinA-7-glucuronide and wogonoside. Comparing retention times with previous reports, we individually identified compounds **12** and **15** as oroxylinA-7-glucuronide and wogonoside, respectively. Compounds **21** and **22** revealed [M-H]^−^ ion at *m/z* 283.0609 and 283.0606, respectively, and these two compounds showed the same fragment ions at *m/z* 268. The MS/MS fragmentation behaviours were similar to wogonin and oroxylin A, respectively. By comparing the present retention times with those in the previous study, we identified compounds **21** and **22** as wogonin and oroxylin A, respectively.

#### 3.1.3. Triterpene and ginsenosides

##### 3.1.3.1. Triterpene

Compound **20** showed [M-H]^−^ ion at *m/z* 821.3959, and the fragment ion at *m/z* 351 corresponded to [2GluA-H_2_O]^−^. The MS/MS fragmentation behaviour was similar to glycyrrhizin, so we identified compound **20** as glycyrrhizin.

##### 3.1.3.2. Ginsenosides

Compound **10** showed [M-H+HCOOH]^−^ ion at *m/z* 845.4938, and the fragment ions at *m/z* 799, 637 and 475 corresponded to [M-H]^−^, [M-H-(Glu-H_2_O)]^−^ and [M-H-2(Glu-H_2_O)]^−^, respectively. The MS/MS fragmentation behaviours were similar to ginsenoside Rf. Compound **16** showed [M-H]^−^ ion at *m/z* 1107.5951, and the fragment ion at *m/z* 945 corresponded to [M-H-(Glu-H_2_O)]^−^. The fragmentation behaviours were similar to those of ginsenoside Rb1; thus, we identified compound **16** as ginsenoside Rb1. Compounds **17** and **18** individually showed [M-H+HCOOH]^−^ at *m/z* 1123.5824 and 1123.5859, and these two compounds showed same fragment ions at *m/z* 1077. According to our previous study, compounds **17** and **18** could be identified as ginsenoside Rc and ginsenoside Rb2, respectively. Compound **19** showed [M-H+HCOOH]^−^ at *m/z* 991.5646, and the fragment ions at *m/z* 945 and 783 corresponded to [M-H]^−^ and [M-H-(Glu-H_2_O)]^−^. The MS/MS fragmentation behaviours were similar to those of ginsenoside Rd. By combining the accurate mass, we identified this compound as ginsenoside Rd.

### 3.2. Network pharmacologic analysis

#### 3.2.1. Compound–compound target network analysis

**Figure 1.**
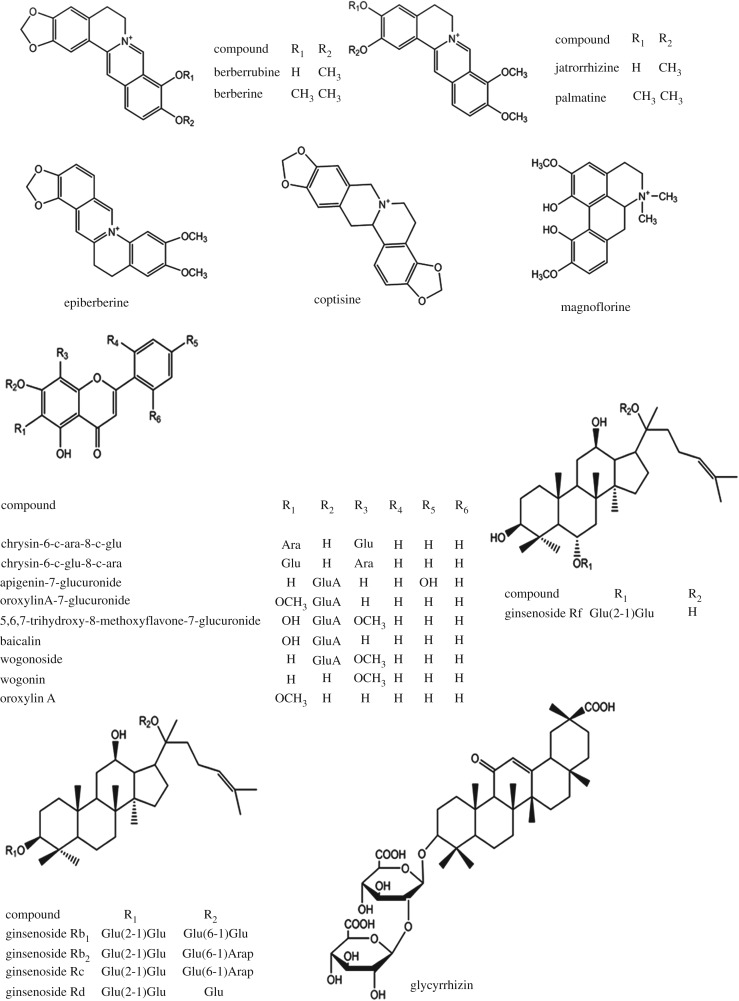
The structures of absorbed components in rat plasma.

**Figure 2.**
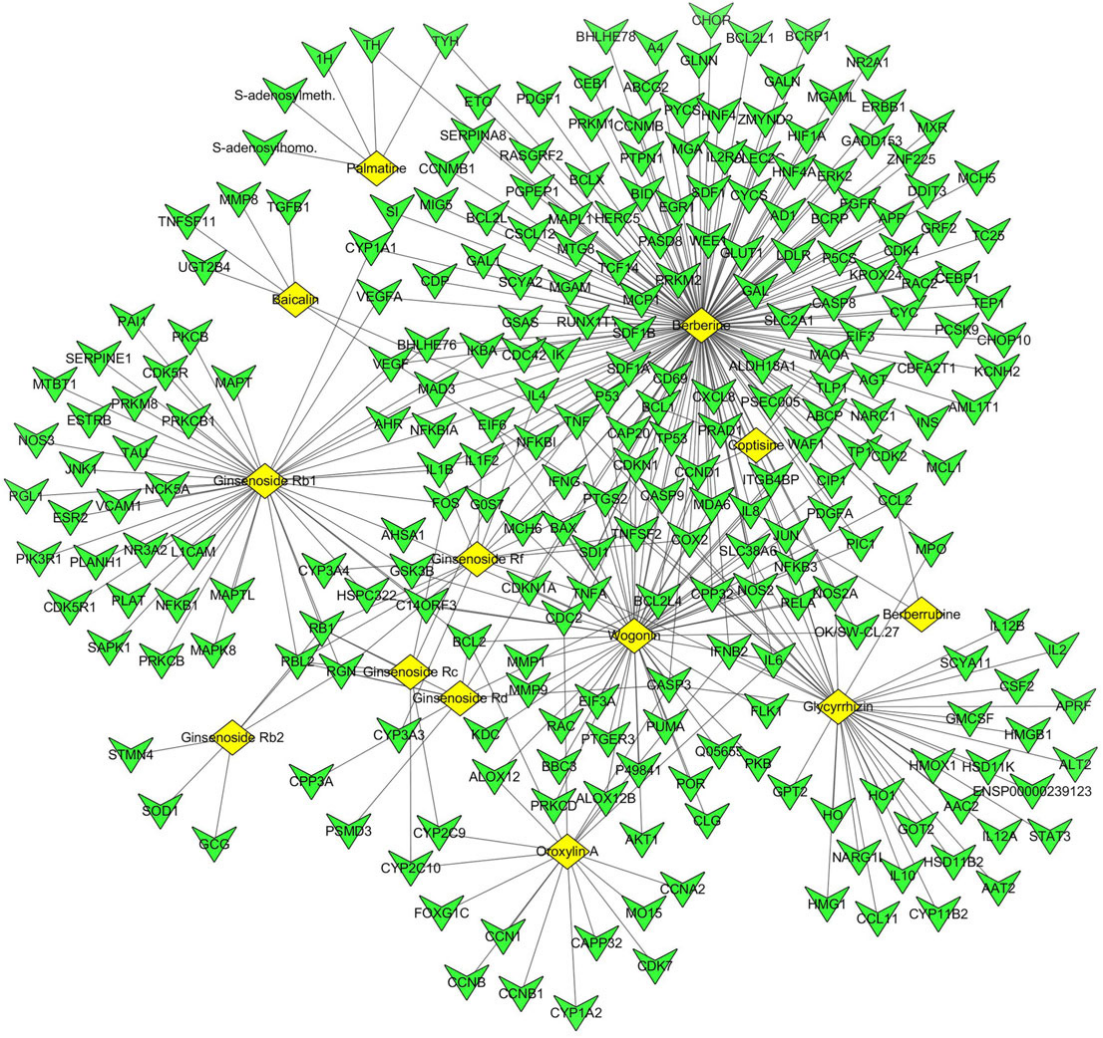
Compound–compound target network of *FXL* (yellow rhombuses stand for the absorbed components, green triangles stand for the compound targets).

The network in figure 1 composed of 272 nodes and 327 edges (figure 2), and the nodes included 259 compound target nodes and 13 compound nodes. Some compound nodes, such as berberine, wogonin, glycyrrhizin and ginsenoside Rb1, in the network achieved a higher degree than that of the other compounds. This result indicated that these compounds may play important roles in treating CAG. From the network, we also found that most targets are only reached by one compound. However, some targets, such as tumour necrosis factor (TNF), PTGS2, IL6 and BCL2, could be aimed by multiple compounds. This result indicated that the targets are closely related to the development of CAG, and these targets are key targets for CAG treatment by *FXL*. For example, TNF can upregulate the expression of COX2 and promote the development of CAG. The compounds related to TNF such as berberine, glycyrrhizin, baicalin, wogonin and ginsenoside Rf, these associated compounds may block the TNF signal and inhibit the upregulation of COX2 expression through synergistic effects and achieve CAG treatment. The treatment mechanism of CAG through *FXL* not only could be clarified, but the pharmacodynamic material basis could also be expounded based on this network.

#### 3.2.2. *Fufang-Xialian-Capsule*-chronic atrophic gastritis target network analysis

**Figure 3.**
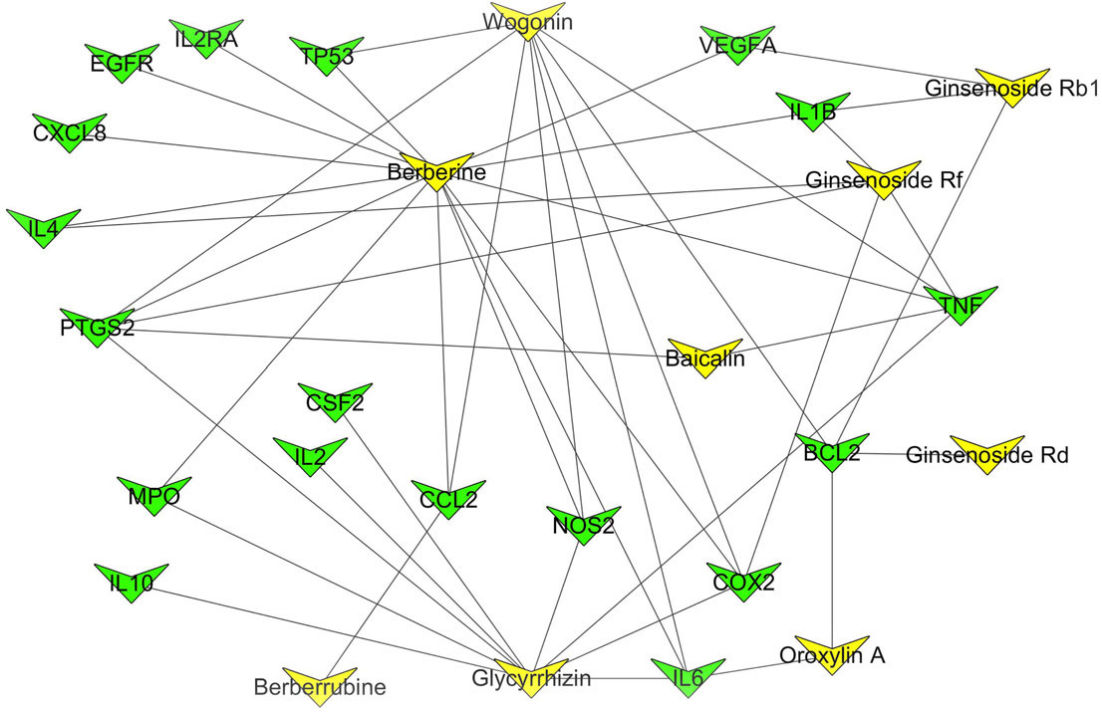
*FXL*-CAG target network (yellow triangles stand for the pharmacodynamic material of *FXL*, green triangles stand for the targets of CAG which relate with the compound targets).

Some compound–compound targets associated with CAG were found by integrating the CAG targets and the compound–compound target (electronic supplementary material, table S3). The network of *FXL*-CAG targets (figure 3) was structured based on these results. The network was composed of 27 nodes (nine compound nodes and 18 target nodes) and 45 edges. In this network, the compound nodes with higher degree than others were similar to the compound–compound target network, such as berberine, wogonin and glycyrrhizin. The results are consistent with our predication on the main functional components of *FXL*.

#### 3.2.3. Target–pathway network analysis

**Figure 4.**
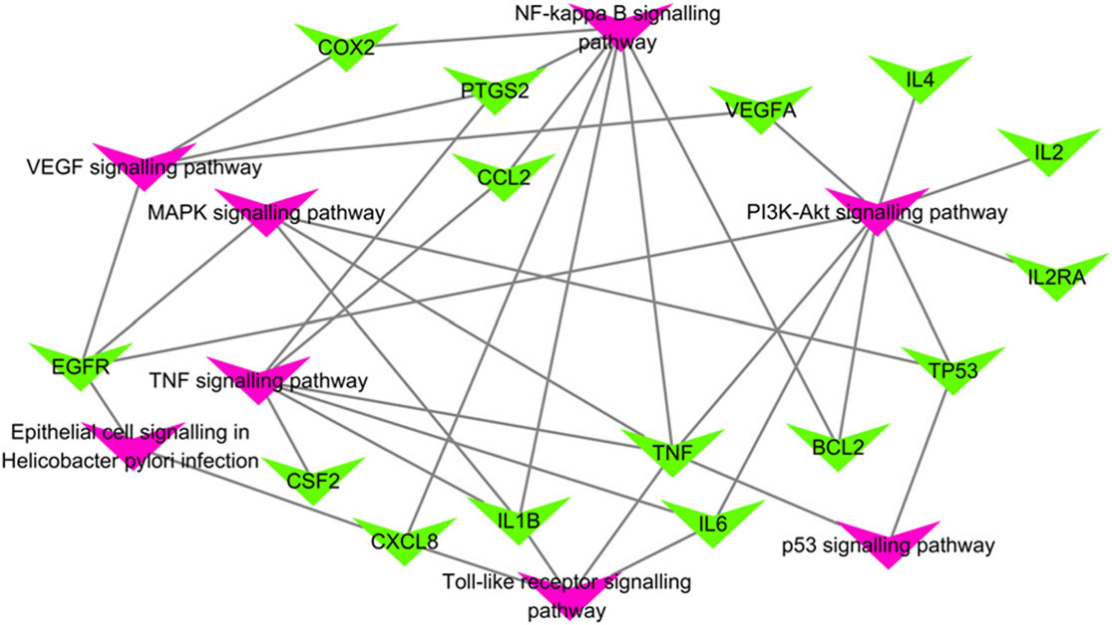
*FXL*-CAG network pathways (green triangles stand for the targets of CAG which relate with the compound targets, pink triangles stand for the pathways related with CAG).

A network including 23 nodes (16 target nodes and eight pathway nodes) and 34 edges (electronic supplementary material table S4) was constructed. A total of eight pathways, which are associated with the treatment of *FXL* on CAG, were obtained by inputting the above genes associated with CAG into DAVID and KEGG. These pathways are the NF-kappa B signalling pathway, TNF signalling pathway, Toll-like receptor signalling pathway, PI3 K-Akt signalling pathway, epithelial cell signalling in *H. pylori* infection, MAPK signalling pathway, p53 signalling pathway and vascular endothelial growth factor (VEGF) signalling pathway (figure 4).

*Helicobacter pylori* infection is the main risk factor for CAG [21], which can damage or kill the gastric epithelial cells and alter the gastric acid secretion [22]. Previous reports have shown that berberine exerts a clearance effect and reduces gastric inflammation during epithelial cell signalling in *H. pylori* infection [23]. Based on this network, the targets associated with epithelial cell signalling are EGFR and CXCL8, which are important responses to *H. pylori* infection [24–26]. *Helicobacter pylori* infection may upregulate the expression of EGFR and CXCL8 through the cell response to epidermal growth factor stimulus (GO 0071364) and the response to bacterial molecules (GO 0002237), respectively. Berberine may act on these two targets to inhibit the CAG development caused by *H. pylori* infection.

Based on the network, the targets associated with the VEGF signalling pathway are EGFR, COX2, PTGS2 and VEGFA. The VEGF signalling can upregulate the expression of COX2, which plays an important role in the development of gastric mucosal lesions [27]. VEGFA and PTGS2 can upregulate the expression of VEGF through the positive regulation of VEGF receptor signalling pathway (GO 0039304) and the positive regulation of VEGF production (GO 0010575). They can also upregulate the expression of COX2. The expression of COX2 can also be upregulated by the NF-kappa B signalling pathway [28]. The other associated targets of the NF-kappa B signalling pathway are PTGS2, CCL2, CXCL8, IL1B, TNF and BCL2. PTGS2 upregulates the expression of COX2 by positively regulating NF-kappa B importing into the nucleus (GO: 0042346). TNF and IL1B can upregulate the expression of COX2 by positively regulating NF-kappa B transcription factor activity (GO: 0051092), as well as regulating I-kappa B kinase/NF-kappa B signalling (GO: 0043122) and positively regulating NF-kappa B transcription factor activity (GO: 0051092). Previous report has shown that wogonin decreases the expression of COX2 [29], because it can decrease VEGF secretion and block the activity of VEGF signalling [30]. Berberine can also inhibit VEGF signalling and NF-kappa B signalling to decrease the expression of COX2 [31].

The MAPK signalling pathway plays an important role in the development of CAG, and it is associated with gastric cancer caused by CAG [32]. Based on the network, the targets associated with MAPK signalling pathway are TNF, IL1B, EGFR and TP53. EGFR can activate MAPK signalling by activating MAPKK activity (GO: 000186) and MAPK cascade (GO: 0000165). TNF can affect MAPK signalling by activating MAPK activity (GO: 0000187), MAPKKK activity (GO: 0000185) and MAPK cascade (GO: 0000165). A previous report has shown that berberine can inactivate MAPK and then inhibit the MAPK signalling pathway [33]. The targets associated with the MAPK signalling pathway may serve as the possible drug targets of berberine, which play an essential role in regulating the MAPK pathway.

The TNF signalling pathway also plays an important role in CAG because it can affect the expression of PTGS2 by responding to TNF (GO: 0034612) and then upregulating the expression of COX2. The other targets, which is associated with the TNF signalling pathway, are CCL2, TNF, IL1B and CSF2. TNF can upregulate the TNF signalling pathway by controlling the TNF-mediated signalling pathway (GO: 0010803). CCL2 can promote the production of TNF by positively regulating TNF production (GO: 0032760) and then upregulating the TNF signalling pathway. All these actions can upregulate the expression of COX2 and affect the development of CAG.

The Toll-like receptor signalling pathway is an important factor of gastric cancer caused by CAG [34]. The PI3 K-Akt signalling pathway is also an important pathway associated with the development of CAG from *H. pylori* infection [35]. The targets associated with the Toll-like receptor signalling pathway are CXCL8, IL1B, TNF and IL6. From the network, we found that the PI3 K-Akt pathway had a higher degree than the other pathways (*n* = 8), and the associated targets were EGRF, VEGF, IL4, IL2, IL2RA, TP53, BCL2, IL6 and TNF. Although the mechanisms of these two pathways and their associated targets in CAG development are unclear, these targets are all possible drug targets for CAG treatment. The other pathways all possess targets with unclear mechanisms, and no report exists on how the associated compounds affect these targets. For example, in these networks, we found that some compounds, such as ginsenoside Rb1, baicalin and oroxylin A, can influence the NF-kappa B signalling pathway and VEGF signalling pathway. The targets associated with baicalin are TNF and PTGS2; baicalin can also affect the NF-kappa B signalling pathway and VEGF signalling pathway by regulating these targets. These targets may serve as the possible drug targets in CAG treatment.

## 4. Conclusion

In this research, the potential pharmacodynamic material basis and pharmacologic mechanism of *FXL* for CAG treatment were investigated via a new network pharmacology approach based on the absorbed components into the blood. The results showed that the components, such as berberine, wogonin and glycyrrhizin, may play important roles in CAG treatment. The possible targets of these compounds were found using the network pharmacology approach. The drug targets in this approach were found to be more directly based on the absorbed components than the targets in the traditional network pharmacology approach. By combining the results of the compound–compound target network, the *FXL*-CAG target network and the *FXL*-CAG network pathways, we clarified the possible treatment mechanism of *FXL* for CAG. Notably, the actions of *FXL* treatment for some compounds, such as berberine and wogonin, can be understood quite well, but the mechanisms of *FXL* treatment for most other compounds and their associated targets remain unclear. However, this research reveals the potential pharmacologic material and the associated pathways, which can provide a basis for further study on the potential pharmacologic and molecular mechanism of *FXL* in CAG treatment. This new method can be used to analyse other Chinese formulae.

## Supplementary Material

Table S1

## Supplementary Material

Table S2

## Supplementary Material

Table S3

## Supplementary Material

Table S4

## Data accessibility

Our data are deposited at Dryad: http://dx.doi.org/10.5061/dryad.g082q0b.
