# Supplementary material for: Exploring the potential pharmacodynamic material basis and pharmacologic mechanism of the Fufang-Xialian-Capsule in chronic atrophic gastritis by network pharmacology approach based on the components absorbed into the blood
Source: R Soc Open Sci. 2018 Jun 13;5(6):171806. doi: 10.1098/rsos.171806 (PMC6030346; doi:10.1098/rsos.171806)
Supplement: Table S1 [file rsos171806supp1.docx]

**Table S1 Compound targets of FXL**

| **Compound** | **Target** |
| --- | --- |
| Berberrubine | CCL2 |
| Berberrubine | IL8 |
| Palmatine | 1H |
| Palmatine | S-adenosylmeth. |
| Palmatine | S-adenosylhomo. |
| Palmatine | TH |
| Palmatine | TYH |
| Berberine | SLC38A6 |
| Berberine | PGPEP1 |
| Berberine | LDLR |
| Berberine | IK |
| Berberine | KCNH2 |
| Berberine | MCL1 |
| Berberine | PTPN1 |
| Berberine | TNF |
| Berberine | TNFA |
| Berberine | TNFSF2 |
| Berberine | CCL2 |
| Berberine | MCP1 |
| Berberine | SCYA2 |
| Berberine | IL6 |
| Berberine | IFNB2 |
| Berberine | CSCL12 |
| Berberine | SDF1 |
| Berberine | SDF1A |
| Berberine | SDF1B |
| Berberine | ABCG2 |
| Berberine | ABCP |
| Berberine | BCRP |
| Berberine | BCRP1 |
| Berberine | MXR |
| Berberine | GAL |
| Berberine | GAL1 |
| Berberine | GALN |
| Berberine | GLNN |
| Berberine | HNF4A |
| Berberine | HNF4 |
| Berberine | NR2A1 |
| Berberine | TCF14 |
| Berberine | INS |
| Berberine | RASGRF2 |
| Berberine | GRF2 |
| Berberine | CDC42 |
| Berberine | RAC2 |
| Berberine | TC25 |
| Berberine | MIG5 |
| Berberine | TP53 |
| Berberine | P53 |
| Berberine | CDKN1A |
| Berberine | CAP20 |
| Berberine | CDKN1 |
| Berberine | CIP1 |
| Berberine | MDA6 |
| Berberine | PIC1 |
| Berberine | SDI1 |
| Berberine | WAF1 |
| Berberine | CDK2 |
| Berberine | CDK4 |
| Berberine | RUNX1T1 |
| Berberine | AML1T1 |
| Berberine | CBFA2T1 |
| Berberine | CDP |
| Berberine | ETO |
| Berberine | MTG8 |
| Berberine | ZMYND2 |
| Berberine | HERC5 |
| Berberine | CEB1 |
| Berberine | CEBP1 |
| Berberine | EIF6 |
| Berberine | EIF3 |
| Berberine | ITGB4BP |
| Berberine | OK/SW-CL.27 |
| Berberine | PCSK9 |
| Berberine | NARC1 |
| Berberine | PSEC005 |
| Berberine | RELA |
| Berberine | NFKB3 |
| Berberine | BID |
| Berberine | JUN |
| Berberine | BCL2L1 |
| Berberine | BCL2L |
| Berberine | BCLX |
| Berberine | CASP3 |
| Berberine | CPP32 |
| Berberine | CASP8 |
| Berberine | MCH5 |
| Berberine | ALDH18A1 |
| Berberine | GSAS |
| Berberine | P5CS |
| Berberine | PYCS |
| Berberine | DDIT3 |
| Berberine | CHOP |
| Berberine | CHOP10 |
| Berberine | GADD153 |
| Berberine | TEP1 |
| Berberine | TLP1 |
| Berberine | TP1 |
| Berberine | MAPL1 |
| Berberine | ERK2 |
| Berberine | PRKM1 |
| Berberine | PRKM2 |
| Berberine | SLC2A1 |
| Berberine | GLUT1 |
| Berberine | APP |
| Berberine | A4 |
| Berberine | AD1 |
| Berberine | IL1B |
| Berberine | IL1F2 |
| Berberine | NOS2 |
| Berberine | NOS2A |
| Berberine | BAX |
| Berberine | BCL2L4 |
| Berberine | CASP9 |
| Berberine | MCH6 |
| Berberine | AGT |
| Berberine | SERPINA8 |
| Berberine | EGFR |
| Berberine | ERBB1 |
| Berberine | EGR1 |
| Berberine | KROX24 |
| Berberine | ZNF225 |
| Berberine | FOS |
| Berberine | G0S7 |
| Berberine | PDGFA |
| Berberine | PDGF1 |
| Berberine | CCNMB1 |
| Berberine | CCNMB |
| Berberine | WEE1 |
| Berberine | CDC2 |
| Berberine | CYCS |
| Berberine | CYC |
| Berberine | NFKBIA |
| Berberine | IKBA |
| Berberine | MAD3 |
| Berberine | NFKBI |
| Berberine | CYP1A1 |
| Berberine | AHR |
| Berberine | BHLHE76 |
| Berberine | CD69 |
| Berberine | CLEC2C |
| Berberine | IL2RA |
| Berberine | VEGFA |
| Berberine | VEGF |
| Berberine | HIF1A |
| Berberine | BHLHE78 |
| Berberine | PASD8 |
| Berberine | PTGS2 |
| Berberine | COX2 |
| Berberine | IL4 |
| Berberine | IFNG |
| Berberine | MGAM |
| Berberine | MGA |
| Berberine | MGAML |
| Berberine | SI |
| Berberine | IL8 |
| Berberine | CXCL8 |
| Berberine | MPO |
| Berberine | TH |
| Berberine | TYH |
| Berberine | CCND1 |
| Berberine | BCL1 |
| Berberine | PRAD1 |
| Berberine | MAOA |
| Coptisine | CCND1 |
| Coptisine | BCL1 |
| Coptisine | PRAD1 |
| Coptisine | MAOA |
| Ginsenoside Rf | RBL2 |
| Ginsenoside Rf | RGN |
| Ginsenoside Rf | RB1 |
| Ginsenoside Rf | IL4 |
| Ginsenoside Rf | PTGS2 |
| Ginsenoside Rf | COX2 |
| Ginsenoside Rf | IL1B |
| Ginsenoside Rf | IL1F2 |
| Ginsenoside Rf | TNF |
| Ginsenoside Rf | TNFA |
| Ginsenoside Rf | TNFSF2 |
| Ginsenoside Rf | IFNG |
| Ginsenoside Rf | CYP3A4 |
| Ginsenoside Rf | CYP3A3 |
| Ginsenoside Rb1 | FOS |
| Ginsenoside Rb1 | RB1 |
| Ginsenoside Rb1 | RBL2 |
| Ginsenoside Rb1 | PIK3R1 |
| Ginsenoside Rb1 | RGN |
| Ginsenoside Rb1 | RGL1 |
| Ginsenoside Rb1 | AHR |
| Ginsenoside Rb1 | BHLHE76 |
| Ginsenoside Rb1 | ESR2 |
| Ginsenoside Rb1 | ESTRB |
| Ginsenoside Rb1 | NR3A2 |
| Ginsenoside Rb1 | GSK3B |
| Ginsenoside Rb1 | CYP1A1 |
| Ginsenoside Rb1 | BCL2 |
| Ginsenoside Rb1 | AHSA1 |
| Ginsenoside Rb1 | C14ORF3 |
| Ginsenoside Rb1 | HSPC322 |
| Ginsenoside Rb1 | MAPK8 |
| Ginsenoside Rb1 | JNK1 |
| Ginsenoside Rb1 | PRKM8 |
| Ginsenoside Rb1 | SAPK1 |
| Ginsenoside Rb1 | NFKBIA |
| Ginsenoside Rb1 | IKBA |
| Ginsenoside Rb1 | MAD3 |
| Ginsenoside Rb1 | NFKB1 |
| Ginsenoside Rb1 | VCAM1 |
| Ginsenoside Rb1 | L1CAM |
| Ginsenoside Rb1 | PRKCB |
| Ginsenoside Rb1 | PKCB |
| Ginsenoside Rb1 | PRKCB1 |
| Ginsenoside Rb1 | SERPINE1 |
| Ginsenoside Rb1 | PAI1 |
| Ginsenoside Rb1 | PLANH1 |
| Ginsenoside Rb1 | PLAT |
| Ginsenoside Rb1 | NOS3 |
| Ginsenoside Rb1 | MAPT |
| Ginsenoside Rb1 | MAPTL |
| Ginsenoside Rb1 | MTBT1 |
| Ginsenoside Rb1 | TAU |
| Ginsenoside Rb1 | CDK5R1 |
| Ginsenoside Rb1 | CDK5R |
| Ginsenoside Rb1 | NCK5A |
| Ginsenoside Rb1 | VEGFA |
| Ginsenoside Rb1 | VEGF |
| Ginsenoside Rb1 | IL1B |
| Ginsenoside Rb1 | IL1F2 |
| Ginsenoside Rc | RGN |
| Ginsenoside Rc | RBL2 |
| Ginsenoside Rc | RB1 |
| Ginsenoside Rc | FOS |
| Ginsenoside Rc | G0S7 |
| Ginsenoside Rc | CYP2C9 |
| Ginsenoside Rc | CYP2C10 |
| Ginsenoside Rb2 | SOD1 |
| Ginsenoside Rb2 | GCG |
| Ginsenoside Rb2 | RBL2 |
| Ginsenoside Rb2 | RGN |
| Ginsenoside Rb2 | STMN4 |
| Ginsenoside Rb2 | RB1 |
| Ginsenoside Rd | RB1 |
| Ginsenoside Rd | RBL2 |
| Ginsenoside Rd | RGN |
| Ginsenoside Rd | CASP3 |
| Ginsenoside Rd | CPP3A |
| Ginsenoside Rd | PSMD3 |
| Ginsenoside Rd | BCL2 |
| Ginsenoside Rd | BAX |
| Ginsenoside Rd | BCL2L4 |
| baicalin | TNF |
| baicalin | PTGS2 |
| baicalin | TNFSF11 |
| baicalin | MMP8 |
| baicalin | TGFB1 |
| baicalin | UGT2B4 |
| Wogonin | IL6 |
| Wogonin | SLC38A6 |
| Wogonin | TNF |
| Wogonin | CCL2 |
| Wogonin | MMP9 |
| Wogonin | POR |
| Wogonin | IL8 |
| Wogonin | ALOX12 |
| Wogonin | ALOX12B |
| Wogonin | RELA |
| Wogonin | NFKB3 |
| Wogonin | CCND1 |
| Wogonin | BCL1 |
| Wogonin | PRAD1 |
| Wogonin | PRKCD |
| Wogonin | Q05655 |
| Wogonin | CDKN1A |
| Wogonin | CAP20 |
| Wogonin | CDKN1 |
| Wogonin | CIP1 |
| Wogonin | MDA6 |
| Wogonin | PIC1 |
| Wogonin | SDI1 |
| Wogonin | WAF1 |
| Wogonin | KDC |
| Wogonin | FLK1 |
| Wogonin | AKT1 |
| Wogonin | PKB |
| Wogonin | RAC |
| Wogonin | AHSA1 |
| Wogonin | C14ORF3 |
| Wogonin | HSPC322 |
| Wogonin | EIF6 |
| Wogonin | EIF3A |
| Wogonin | ITGB4BP |
| Wogonin | OK/SW-CL.27 |
| Wogonin | TP53 |
| Wogonin | P53 |
| Wogonin | BBC3 |
| Wogonin | PUMA |
| Wogonin | BAX |
| Wogonin | BCL2L4 |
| Wogonin | CASP9 |
| Wogonin | MCH6 |
| Wogonin | GSK3B |
| Wogonin | P49841 |
| Wogonin | CASP3 |
| Wogonin | CPP32 |
| Wogonin | MMP1 |
| Wogonin | CLG |
| Wogonin | JUN |
| Wogonin | NOS2 |
| Wogonin | NOS2A |
| Wogonin | BCL2 |
| Wogonin | PTGS2 |
| Wogonin | COX2 |
| Wogonin | PTGER3 |
| Oroxylin A | CDC2 |
| Oroxylin A | CCNB1 |
| Oroxylin A | CCNB |
| Oroxylin A | CCNA2 |
| Oroxylin A | CCN1 |
| Oroxylin A | CDK7 |
| Oroxylin A | MO15 |
| Oroxylin A | BCL2 |
| Oroxylin A | CASP3 |
| Oroxylin A | CAPP32 |
| Oroxylin A | CYP2C9 |
| Oroxylin A | CYP2C10 |
| Oroxylin A | CYP1A2 |
| Oroxylin A | IL6 |
| Oroxylin A | IFNB2 |
| Oroxylin A | ALOX12 |
| Oroxylin A | ALOX12B |
| Oroxylin A | FOXG1C |
| glycyrrhizin | ENSP00000239123 |
| glycyrrhizin | GOT2 |
| glycyrrhizin | IL12B |
| glycyrrhizin | IL12A |
| glycyrrhizin | AAC2 |
| glycyrrhizin | NARG1L |
| glycyrrhizin | SLC38A6 |
| glycyrrhizin | IL10 |
| glycyrrhizin | CSF2 |
| glycyrrhizin | GMCSF |
| glycyrrhizin | IL6 |
| glycyrrhizin | IFNB2 |
| glycyrrhizin | IL2 |
| glycyrrhizin | HMGB1 |
| glycyrrhizin | HMG1 |
| glycyrrhizin | RELA |
| glycyrrhizin | NFKB3 |
| glycyrrhizin | STAT3 |
| glycyrrhizin | APRF |
| glycyrrhizin | CASP3 |
| glycyrrhizin | CPP32 |
| glycyrrhizin | TNF |
| glycyrrhizin | TNFA |
| glycyrrhizin | TNFSF2 |
| glycyrrhizin | MPO |
| glycyrrhizin | PTGS2 |
| glycyrrhizin | COX2 |
| glycyrrhizin | HMOX1 |
| glycyrrhizin | HO |
| glycyrrhizin | HO1 |
| glycyrrhizin | NOS2 |
| glycyrrhizin | NOS2A |
| glycyrrhizin | CCL11 |
| glycyrrhizin | SCYA11 |
| glycyrrhizin | JUN |
| glycyrrhizin | CYP11B2 |
| glycyrrhizin | HSD11B2 |
| glycyrrhizin | HSD11K |
| glycyrrhizin | GPT2 |
| glycyrrhizin | AAT2 |
| glycyrrhizin | ALT2 |
