# Supplementary material for: Exploring the potential pharmacodynamic material basis and pharmacologic mechanism of the Fufang-Xialian-Capsule in chronic atrophic gastritis by network pharmacology approach based on the components absorbed into the blood
Source: R Soc Open Sci. 2018 Jun 13;5(6):171806. doi: 10.1098/rsos.171806 (PMC6030346; doi:10.1098/rsos.171806)
Supplement: Table S2 [file rsos171806supp2.docx]

**Table S2 Genes associated with CAG**

| **NO** | **Symbol** |
| --- | --- |
| 1 | IL1B |
| 2 | IL1RN |
| 3 | CXCL8 |
| 4 | TNF |
| 5 | OGG1 |
| 6 | IL10 |
| 7 | PTPN11 |
| 8 | TP53 |
| 9 | LTA |
| 10 | PGC |
| 11 | CDX2 |
| 12 | PTGS2 |
| 13 | SHH |
| 14 | TGFA |
| 15 | HLA-DRB1 |
| 16 | IL2 |
| 17 | TLR4 |
| 18 | IL4R |
| 19 | IL13 |
| 20 | PSCA |
| 21 | GAB1 |
| 22 | IL1A |
| 23 | ALDH2 |
| 24 | RUNX3 |
| 25 | AREG |
| 26 | GHRL |
| 27 | CDX1 |
| 28 | TLR2 |
| 29 | AICDA |
| 30 | IL4 |
| 31 | HLA-DQB1 |
| 32 | HLA-DQA1 |
| 33 | PRKCH |
| 34 | MTHFR |
| 35 | ACE |
| 36 | IL6 |
| 37 | MSH2 |
| 38 | MLH1 |
| 39 | MBL2 |
| 40 | XRCC1 |
| 41 | MIF |
| 42 | GSTP1 |
| 43 | ERCC2 |
| 44 | HSPA2 |
| 45 | DNAH8 |
| 46 | S100A8 |
| 47 | GAST |
| 48 | ACP1 |
| 49 | RBM45 |
| 50 | MEN1 |
| 51 | TFF2 |
| 52 | COX2 |
| 53 | GNA12 |
| 54 | ABO |
| 55 | MPO |
| 56 | CD44 |
| 57 | MUC1 |
| 58 | ODC1 |
| 59 | CYP2C19 |
| 60 | GSTM1 |
| 61 | GSTT1 |
| 62 | GRN |
| 63 | MIRLET7A1 |
| 64 | NOX1 |
| 65 | TPO |
| 66 | MIRLET7A2 |
| 67 | ATP11A |
| 68 | MIR146A |
| 69 | ABCB6 |
| 70 | PGA3 |
| 71 | IGHV3-76 |
| 72 | GKN1 |
| 73 | TRAF1 |
| 74 | CLDN18 |
| 75 | SFXN1 |
| 76 | PLCE1 |
| 77 | NOXO1 |
| 78 | DLK1 |
| 79 | VEGFA |
| 80 | PDLIM7 |
| 81 | FLVCR1 |
| 82 | TTF1 |
| 83 | MIRLET7C |
| 84 | TLR1 |
| 85 | CSF2 |
| 86 | GLUL |
| 87 | FUT6 |
| 88 | FOXJ1 |
| 89 | ERCC6 |
| 90 | EGFR |
| 91 | DNMT1 |
| 92 | CYP2E1 |
| 93 | CDKN2A |
| 94 | BCL2 |
| 95 | ATP4A |
| 96 | ATP12A |
| 97 | AIRE |
| 98 | HLA-DQA2 |
| 99 | HLA-DQB2 |
| 100 | NKX2-1 |
| 101 | TCN2 |
| 102 | SPP1 |
| 103 | CCL2 |
| 104 | SERPINA1 |
| 105 | PDC |
| 106 | NOS2 |
| 107 | MDM2 |
| 108 | LEP |
| 109 | KRAS |
| 110 | ISG20 |
| 111 | IL2RA |
| 112 | IFNGR1 |
| 113 | APBA2 |
