# Supplementary material for: Exploring the potential pharmacodynamic material basis and pharmacologic mechanism of the Fufang-Xialian-Capsule in chronic atrophic gastritis by network pharmacology approach based on the components absorbed into the blood
Source: R Soc Open Sci. 2018 Jun 13;5(6):171806. doi: 10.1098/rsos.171806 (PMC6030346; doi:10.1098/rsos.171806)
Supplement: Table S3 [file rsos171806supp3.docx]

**Table S3 CAG target associated with compound target**

| **Compound** | **Target** |
| --- | --- |
| Berberine | IL1B |
| Berberine | CXCL8 |
| Berberine | TNF |
| Berberine | TP53 |
| Berberine | PTGS2 |
| Berberine | IL4 |
| Berberine | IL6 |
| Berberine | COX2 |
| Berberine | MPO |
| Berberine | VEGFA |
| Berberine | EGFR |
| Berberine | CCL2 |
| Berberine | NOS2 |
| Berberine | IL2RA |
| Berberrubine | CCL2 |
| Glycyrrhizin | TNF |
| Glycyrrhizin | IL10 |
| Glycyrrhizin | PTGS2 |
| Glycyrrhizin | IL2 |
| Glycyrrhizin | IL6 |
| Glycyrrhizin | COX2 |
| Glycyrrhizin | MPO |
| Glycyrrhizin | CSF2 |
| Glycyrrhizin | NOS2 |
| Wogonin | TNF |
| Wogonin | TP53 |
| Wogonin | PTGS2 |
| Wogonin | IL6 |
| Wogonin | COX2 |
| Wogonin | BCL2 |
| Wogonin | CCL2 |
| Wogonin | NOS2 |
| Baicalin | TNF |
| Baicalin | PTGS2 |
| Oroxylin A | IL6 |
| Oroxylin A | BCL2 |
| Ginsenoside Rf | IL1B |
| Ginsenoside Rf | TNF |
| Ginsenoside Rf | PTGS2 |
| Ginsenoside Rf | IL4 |
| Ginsenoside Rf | COX2 |
| Ginsenoside Rb1 | IL1B |
| Ginsenoside Rb1 | VEGFA |
| Ginsenoside Rb1 | BCL2 |
| Ginsenoside Rd | BCL2 |
