# Supplementary material for: Exploring the potential pharmacodynamic material basis and pharmacologic mechanism of the Fufang-Xialian-Capsule in chronic atrophic gastritis by network pharmacology approach based on the components absorbed into the blood
Source: R Soc Open Sci. 2018 Jun 13;5(6):171806. doi: 10.1098/rsos.171806 (PMC6030346; doi:10.1098/rsos.171806)
Supplement: Table S4 [file rsos171806supp4.docx]

**Table S4 Pathways with their related targets**

| **Symbol** | **Pathway** |
| --- | --- |
| IL1B | Toll-like receptor signaling pathway |
| IL1B | NF-kappa B signaling pathway |
| IL1B | TNF signaling pathway |
| IL1B | MAPK Signaling pathway |
| CXCL8 | NF-kappa B signaling pathway |
| CXCL8 | Toll-like receptor signaling pathway |
| CXCL8 | Epithelial cell signaling in Helicobacter pylori infection |
| TNF | MAPK Signaling pathway |
| TNF | NF-kappa B signaling pathway |
| TNF | TNF signaling pathway |
| TNF | Toll-like receptor signaling pathway |
| TNF | p53 signaling pathway |
| TNF | PI3K-Akt signaling pathway |
| PTGS2 | NF-kappa B signaling pathway |
| PTGS2 | TNF signaling pathway |
| PTGS2 | VEGF signaling pathway |
| BCL2 | NF-kappa B signaling pathway |
| BCL2 | PI3K-Akt signaling pathway |
| IL6 | TNF signaling pathway |
| IL6 | Toll-like receptor signaling pathway |
| IL6 | PI3K-Akt signaling pathway |
| CSF2 | TNF signaling pathway |
| CCL2 | TNF signaling pathway |
| CCL2 | NF-kappa B signaling pathway |
| TP53 | MAPK Signaling pathway |
| TP53 | p53 signaling pathway |
| TP53 | PI3K-Akt signaling pathway |
| VEGFA | VEGF signaling pathway |
| VEGFA | PI3K-Akt signaling pathway |
| IL2 | PI3K-Akt signaling pathway |
| IL2RA | PI3K-Akt signaling pathway |
| IL4 | PI3K-Akt signaling pathway |
| EGFR | MAPK Signaling pathway |
| EGFR | PI3K-Akt signaling pathway |
| EGFR | VEGF signaling pathway |
| EGFR | Epithelial cell signaling in Helicobacter pylori infection |
| COX2 | NF-kappa B signaling pathway |
| COX2 | VEGF signaling pathway |
